# Promoting Mental Health in Adolescents Through Physical Education: Measuring Life Satisfaction for Comprehensive Development

**DOI:** 10.3390/children12050658

**Published:** 2025-05-21

**Authors:** Santiago Gómez-Paniagua, Antonio Castillo-Paredes, Pedro R. Olivares, Jorge Rojo-Ramos

**Affiliations:** 1 Promoting a Healthy Society Research Group (PHeSO), Faculty of Sport Sciences, University of Extremadura, 10003 Cáceres, Spain; sgomezpa@alumnos.unex.es (S.G.-P.); jorgerr@unex.es (J.R.-R.); 2 Grupo AFySE, Investigación en Actividad Física y Salud Escolar, Escuela de Pedagogía en Educación Física, Facultad de Educación, Universidad de Las Américas, Santiago 8370040, Chile; 3 Faculty of Education, Psychology and Sport Sciences, University of Huelva, 21007 Huelva, Spain; pedro.olivares@ddi.uhu.es; 4 Facultad de Educación, Universidad Autónoma de Chile, Talca 3480094, Chile

**Keywords:** mental health, adolescents, sex, metric invariance, temporal stability

## Abstract

**Background:** Life satisfaction serves as a preventive agent against various emotional, cognitive, and behavioral challenges, making it a crucial cognitive indicator of subjective well-being, particularly during adolescence. Accurately assessing life satisfaction is essential for understanding and promoting adolescent mental health, especially in applied settings such as physical education, which plays a key role in fostering psychological well-being and positive youth development. However, additional investigation is needed to confirm the tools used for this purpose. This study aimed to analyze the psychometric properties, metric invariance, and temporal stability of the Satisfaction with Life Scale (SWLS) in adolescents from a region in southeastern Spain. Thus, the present study sought to answer the following research questions: (1) Does the SWLS demonstrate adequate psychometric properties in an adolescent population? (2) Is the SWLS invariant across gender and residential environments? (3) Does the SWLS show adequate stability over time? **Methods:** A sample of 400 students was assessed using exploratory and confirmatory factor analyses, multigroup comparisons, and test–retest techniques. **Results:** The results showed significant differences in scale scores in the sex and demographic location variables. Also, a robust unifactorial model with five items demonstrated good performance in terms of goodness of fit and internal consistency. Furthermore, full metric invariance was observed across genders, while configural invariance was supported for residential environment. Concurrent validity analyses revealed significant associations with another unidimensional well-being measure, and temporal stability was confirmed through the intraclass correlation coefficient. **Conclusions:** The findings support the SWLS as a potentially valid, reliable, and time-effective tool for assessing adolescent life satisfaction. Its strong psychometric properties make it highly suitable for use in mental health research, longitudinal monitoring, and large-scale studies. Moreover, its ease of administration allows its integration into educational, clinical, community-based, and physical education contexts, offering insightful information for the creation of long-lasting mental health regulations and preventive measures meant to improve the well-being of adolescents. Notwithstanding these encouraging results, some restrictions must be noted. The sample was restricted to a single geographic area, and contextual or cultural factors may have an impact on how satisfied people are with their lives. Furthermore, response biases could have been introduced by using self-report measures.

## 1. Introduction

The emotional response that each person has to their life and the expectations they have for it, considering their perspectives on work, social life, and personal life, as well as their biological and psychological needs, is what is known as life satisfaction (LS) [1,2]. Both LS and quality of life—more especially, leading a healthy lifestyle—are strongly correlated with health [3]. Accordingly, many studies examining the connection between LS and physical activity (PA) have found that decreased amounts of PA are associated with worse health, which in turn causes LS to decline [4]. Conversely, research has shown that adolescents, irrespective of gender, who engage in more frequent and intense physical exercise have higher LS [5,6,7]. In addition, due to biological, psychological, cognitive, and social changes that may impact the LS assessment process during adolescence, self-concept may be seen as a sign of how adolescents manage these changes in relation to LS [8]. This has led to the identification of adolescence as a stage of mental health risk in the life cycle. In keeping with this line of reasoning, children’s and teenagers’ LS appears to be greatly influenced by their educational environment [9]. In other words, higher LS appears to be associated with academic performance, and the most significant mediator for enhancing students’ mental health is physical activity (PA) [10].

In a similar vein, many elements have been found to be significant when examining children’s and adolescents’ SL. In this sense, various variables, such as an individual’s gender, can serve as predictors or modify the effect on LS [11]. Research indicates that when focusing on the childhood and adolescent stages, girls typically have lower SV than boys [12]. In addition, living environments also have a significant impact on people’s LS levels [13], although there is not much research that repeats these kinds of investigations in children and teenagers [14,15]. However, some research indicates that family functioning and positive youth development are linked, over time, to adolescent SV [16]. Other relationships with SV include family structure, family life cycle stage, and nutrition [17]. In the end, it is anticipated that adolescents with SV will be happier with their lives if they believe they are more capable socially and academically, have the capacity for self-control, and have worthwhile goals to work toward, while adolescents from less affluent families exhibit lower levels of LS [18]. On the other hand, a variety of other pathologies, including cardiovascular illnesses and sleep disorders, are linked to childhood and teenage overweight [19], and these disorders’ aftereffects and complications impair children’s and teenagers’ subjective well-being and lower their LS [20,21].

Because of the evident importance of LS as a protective factor in the normal development of children and adolescents, there are numerous assessment tools available for these populations [22]. Among them, the Satisfaction with Life Scale (SWLS) [23] stands out. It has a five-item single-factor structure, and analyzes the general judgment of adolescents regarding their subjective well-being. Moreover, this scale has been translated over time into different languages [24,25,26] and validated in different populations [27,28,29], showing disparate characteristics. The adolescent population has not been an exception, with validations of the scale in different areas around the world, whether in Europe [30,31], Asia [32] or South America [33]. However, as far as Spain is concerned, only Ortuño and colleagues [34] have tested metric invariance in adolescents according to their sociodemographic factors, since other studies that included Spanish adolescents evaluated it by comparing them with their counterparts in other countries [35,36]. Similarly, and to the authors’ knowledge, no study has conducted an invariance analysis focused on the area in which the student lives (rural or urban), which has been pointed out as an influential factor in the LS levels of adolescents [13,14], just as no validation study has included data from teenagers living in the region of Extremadura (Spain). Therefore, the current study’s objectives were to investigate the SWLS’s characteristics and conduct an invariance analysis that takes gender and the environment of a sample of adolescents from a region in southeast Spain under consideration. Similarly, possible differences in the study population according to the variables of analysis will be explored in order to understand the current state of the region. Based on consistent one-factor structures in prior adolescent SWLS validations, we hypothesized that the internal structure would be monofactorial, composed of five items, offer good goodness-of-fit indices, and that the assumption of metric invariance would be met for both the gender variable and the environment. Thus, the present study sought to answer the following research questions:

1. Does the SWLS demonstrate adequate psychometric properties in an adolescent population from Extremadura?
2. Is the SWLS invariant across gender and residential environments?
3. Does the SWLS show adequate stability over time?

## 2. Materials and Methods

### 2.1. Participants

Because the goal of this study was to collect as many responses as possible, we used a descriptive cross-sectional design. As per the latest data available on the National Institute of Statistics (www.ine.es), within the Community of Extremadura in Spain, there are 43,043 minors aged 8 to 18. Our study’s sample size of 400 participants was greater than the 381 needed to guarantee a 95% confidence level and a ±5% margin of error. The inclusion criteria for participation in the study were regular participation in PE classes and informed consent from parents.

A total of 400 participants comprised the study’s sample (Table 1), chosen via convenience sampling in accordance with Salkind’s suggested procedures [37], with boys representing the same proportion of the sample (50%, *n* = 200) as girls (50%, *n* = 200). When it came to geographic location, 45% (*n* = 180) of the population lived in rural areas, while the majority (55%, *n* = 220) lived in urban areas. With a mean age of 13.12 years and a standard deviation of 1.94, the group’s age variability was deemed to be moderate.

In this study, living environments were categorized using the Cáceres Provincial Council’s concept. Urban communities were defined as having 20,000 or more inhabitants, whereas rural communities had 20,000 or fewer.

The University of Extremadura’s Ethics Committee gave its approval to the study (6/2024), which was carried out in compliance with the Declaration of Helsinki’s criteria.

### 2.2. Procedure

Emails with information on the study, a sample questionnaire, and a request for parental approval were sent to Extremadura’s physical education teachers. A list of local public schools offering secondary education was used to choose the teachers. The teachers in question arranged for a member of the study team to visit the center and provide the questionnaires to the pupils after getting the families’ signed agreement. A week later, the process would be repeated.

Every student received a tablet from the researcher on the scheduled day, along with a link to the Google Forms questionnaire. The researcher read out each question to make sure the participants understood it. To enable effective data collection and storage, it was decided to employ an electronic questionnaire.

The questionnaire took about five minutes to complete, and anonymous data were gathered between January and February of 2023.

### 2.3. Instruments

Initially, a set of three sociodemographic questions about sex, housing environment, and age were included in the questionnaire.

Additionally, the Satisfaction with Life Scale (SWLS) (Appendix A), which Atienza and collaborators validated in Spanish [26], was used to gauge teenagers’ subjective levels of LS. This instrument has five items that are intended to gauge young people’s values for various elements of their lives. A Likert-type scale, with 1 denoting “strongly disagree” and 5 denoting “strongly agree”, is used to collect responses. The scale’s authors reported a Cronbach’s alpha coefficient of 0.84, demonstrating the scale’s good reliability when gauging adolescent’ LS. This scale gathers items such as “In most aspects, my life is the way I want it to be” or “I am satisfied with my life”.

Lastly, the six-level Student’s Life Satisfaction Scale (SLSS) in Spanish (Appendix B) was utilized. It was validated by Alfaro et al. [38] and had a Cronbach’s alpha score of 0.70. Items 3 and 4 were inverted to make it easier to interpret the results. The scale ranged from “strongly disagree” (1) to “strongly agree” (6).

### 2.4. Statistical Analysis

The SWLS items and overall scores were analyzed using descriptive statistics. The normality of the data was evaluated using the Kolmogorov–Smirnov test. The Mann–Whitney U test was used to analyze differences by sex and living environment in light of the non-normal distribution. Hedges’ g was used to calculate effect sizes.

The Solomon approach was used to randomly split the sample into two subsamples in order to examine the psychometric characteristics of the SWLS. One subsample was subjected to exploratory factor analysis (EFA) using the software FACTOR v.10.10.02, while the other was subjected to confirmatory factor analysis (CFA) using AMOS v.26.0.0. Sample adequacy was evaluated using the Kaiser–Meyer–Olkin (KMO) metric and Bartlett’s test of sphericity.

Also, multigroup CFA was used to examine measurement invariance across groups (gender and residential environment). Spearman correlation with a well-being scale that had been validated in Spanish adolescents (SLSS) was used to assess concurrent validity.

Intraclass correlation coefficients (ICCs) with 95% CIs were used to evaluate temporal stability and test–retest reliability. Additionally, the minimal detectable change (MDC) and the standard error of measurement (SEM) were computed.

## 3. Results

### 3.1. Descriptive Statistics and Differences

The descriptive statistics and differences for each of the items that make up the SWLS questionnaire by gender and setting are presented in Table 2. In terms of effect size, values below 0.20 are considered to have no effect, a modest influence for coefficients between 0.21 and 0.49, a moderate effect for values between 0.50 and 0.79, and a substantial effect for values above 0.80 [39].

Looking at the gender variable, male students showed statistically significantly higher scores on all items of the questionnaire. However, items 1 and 4 showed insignificant effect sizes, although the rest of the items and the total score showed modest effects. Moreover, some item-level differences were observed by residential environment, with urban students showing higher scores in items one, two and five, although in items three and four, rural students obtained a higher mean score. The sole item that also revealed statistically significant differences favoring students from rural contexts was item 4, indicating a modest effect of these differences.

### 3.2. EFA and CFA

First, the sample was divided into two equal subsamples using Solomon’s method [40], one for the EFA and the other for the CFA. This allows analysis of whether the model explored in one subsample (EFA) is replicated in a second (CFA), establishing a procedure in which all possible sources of common variance are equally represented in each subsample. The RULS method enabled the identification of a unidimensional structure for the questionnaire (Table 3), supported by the amount of variance explained through eigenvalues [41] and the reliability estimates obtained via expected a posteriori (EAP) scores [42].

Due to the outcome, no rotation mechanism was selected because the structure was one-dimensional. The feasibility of conducting the EFA was confirmed through favorable sample adequacy metrics, with a KMO value of 0.834 and a statistically significant Bartlett’s test result (χ^2^ = 551.3; df = 10; *p* < 0.001). Table 4 presents the factor loading matrix for a one-factor solution comprising five items.

**Figure 1:**
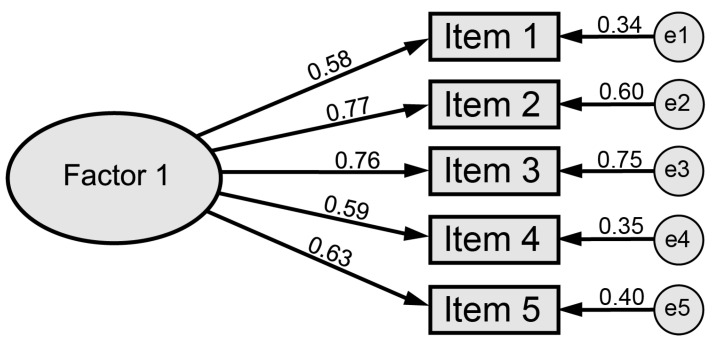
Factorial structure.

Once the EFA confirmed the structure of the scale, a CFA was conducted using the second subsample to assess the model’s characteristics (see Figure 1). All items were retained, as they satisfied the established criteria: absence of cross-loadings above 0.40, communalities exceeding 0.30, and factor loadings equal to or greater than 0.60.

The model’s goodness-of-fit indices were found to be satisfactory, even though the factorial structure was maintained without introducing correlations between the error terms of items 4 and 5, as suggested in prior studies [25]. To assess model fit, several indicators were considered: the chi-squared-to-degrees of freedom ratio (CMIN/DF) with a threshold below 3 [43]; the root mean square error of approximation (RMSEA) and the root mean square of residuals (RMSR), both expected to be under 0.08 [44,45]; and comparative (CFI) and normed fit indices (NFI), with recommended values above 0.90 [46].

In this analysis, the CMIN/DF reached a value of 2.17 (χ^2^ = 10.86, df = 5), while the CFI and NFI were 0.98 and 0.97, respectively. Additionally, the RMSEA and RMSR values were 0.07 and 0.03, indicating an adequate model fit. Table 5 displays the standardized factor loadings by group.

### 3.3. Measurement Invariance

Likewise, multiple multigroup confirmatory factor analyses were performed to assess measurement invariance across groups. To determine whether the nested models demonstrated invariance, a change in the comparative fit index (CFI) of less than 0.01 was used as the evaluation criterion [47].

The fit indices obtained from the successive multigroup analyses revealed a variation of less than 0.01 between the unconstrained and constrained models, thereby supporting the presence of measurement invariance across both variables (Table 6).

### 3.4. Internal Consistency, Concurrent Validity, and Temporal Stability

Furthermore, as measures of scale reliability, McDonald’s omega and Cronbach’s alpha were employed [48]. As far as internal consistency is concerned, the scale showed acceptable values in both measurement times: Cronbach’s alpha (time 1 = 0.83, time 2 = 0.82) and McDonald’s omega (time 1 = 0.83, time 2 = 0.82).

In the same vein, the analysis of the relationship between the SWLS and SLSS scores indicated a statistically significant and direct correlation of moderate magnitude (ρ = 0.60, *p* < 0.001).

Finally, in order to assess the temporal stability of the SWLS, test–retest reliability was assessed using the intraclass correlation coefficient (ICC) between the two measurement times with 95% confidence interval. These results were classified by the intervals defined by Cicchetti [49]: poor (<0.40), fair (0.40–0.60), good (0.60–0.75), or excellent (>0.75). In accordance with recommendations for selecting the ICC [50], it was based on two-way random effects, utilizing the mean of multiple measurements and absolute agreement. Furthermore, calculations were made for the minimal detectable change (MDC) and standard error of measurement (SEM) [51,52].

The values related to temporal stability are shown in Table 7. The ICC between 0.6 and 0.75 showed good reliability of the questionnaire in the two data collections.

## 4. Discussion

The primary objective of this study was to evaluate the psychometric properties of the SWLS in a population of adolescents belonging to a region of southeastern Spain (Extremadura), as well as to confirm its metric invariance according to gender and the environment where they live. Likewise, an update of the current state of LS of adolescents in the region was produced, finding differences in the sex variable and in the area of residence.

As for the differences found in gender, the most current literature exploring SL at the international level in some thirty countries shows that in general terms, female adolescents show lower levels compared to their male peers [53]. In the Spanish context, Aymerich et al. [54] conducted a retrospective study in 600 Spanish adolescents, identifying female students as the most vulnerable, with the most dangerous period being from 10 to 12 years of age. Likewise, in a sample of 2400 adolescents aged 12 to 17 years in southern Spain, Reina Flores et al. [55] found similar results. However, Casas Aznar et al. [56] found positive differences favoring females in a large number of adolescents enrolled in secondary education. As researchers note [57], these discrepancies could be due to females having more social support, but revealing negative emotions more often than men in daily life, causing LS levels to level out over time.

To the best of the authors’ knowledge, there is a dearth of scientific research examining how adolescent behavior varies depending on their place of residence. Overall, Márquez and Long [58] assessed LS levels in 15-year-old adolescents from 46 nations and discovered that in spite of the recent reduction, pupils from rural settings had higher levels. Similar findings were reported in an Indian study that examined university students’ LS in this setting, which explained why urban students performed worse because of their busy schedules and stress accumulation [59]. However, the latest scientific publications indicate that the trend is towards equality, finding similar scores for students in both contexts, although this varies depending on the society being studied [60]. Numerous psychosocial reasons could be the cause of the lack of notable variances. For instance, adolescents living in urban settings may experience higher levels of academic anxiety, lower feelings of belonging at school, and more exposure to victimization, insecurity, and family conflict, all of which are linked to behavioral problems and poorer psychological well-being [61,62,63,64,65,66]. Potential urban benefits, including greater access to services or educational opportunities, may be offset by these pressures. According to Jiménez Boraita et al. [67] and Sharma et al. [59], rural youth’s reported LS may also be influenced by their greater levels of health-related well-being and longer sleep duration.

In this sense, the results confirmed adequate values in the sample of Spanish-speaking adolescents, as other studies had previously ratified [34,68]. This study established a unifactorial structure composed of five items that yielded good goodness-of-fit indices, like other international studies [69,70], though despite the improvement in these indices, the correlation of items four and five was not necessary to statistically define the model, as recent studies pointed out [32,71]. Likewise, the reliability values obtained are in agreement with previous studies carried out on samples of adolescents in the same country [34,35].

Regarding metric invariance, numerous studies have tested the gender variable. In this sense, Emerson and collaborators have already indicated that gender bias in the questionnaire was not a factor to be taken into account [72]. More recent studies focusing on adolescent populations have confirmed this assertion: in France [73], India [32], and Peru or Portugal [74]. Likewise, Jovanović et al. [71] carried out a study of the scale in 24 countries, asserting the metric invariance of the instrument with respect to gender in 21 of the regions explored. However, they suggested that this invariance should not be taken for granted and needed to be evaluated prior to the application of the questionnaire, mainly due to the complexity of the results obtained.

To the authors’ knowledge, the environment in which the adolescent resides has not been the subject of study in terms of metric invariance. Nevertheless, it has been pointed out as an important limitation in previous research, due to the fact that there are many regions in which the population is evenly distributed between urban and rural areas [75].

Moreover, moderate concurrent validity was obtained when compared with another measurement scale of the same construct, as already demonstrated in previous literature [75,76]; however, there are few studies in which it is compared with another unifactorial measure of LS. On the other hand, temporal stability remains a parameter little analyzed in the different SWLS validations. The vast majority of studies use bivariate correlations to assess the temporal stability of the instrument [32,77], although the statistical literature has pointed out that the ICC is more appropriate for assessing the temporal stability of measures associated with health-related quality of life because the measures to be compared at both instants are randomly selected [78]. In this context, Silva et al. [30] found very similar test–retest results to those of this study.

### Limitations and Future Lines

This research presents a series of limitations like any other. First, no data were collected from students at baccalaureate level (from 16 to 18 years old), so LS levels in late adolescence cannot be contrasted. Also, the results of this study should be regarded cautiously primarily because of its design, which makes it impossible to establish case–effect correlations. Furthermore, because it was limited to the community of Extremadura, sociocultural factors could have affected the outcomes, in addition to the convenience sampling technique used to choose the participants. Finally, there are few examples in the scientific literature that explore the temporal stability of the questionnaire and the metric invariance of the selected sample, the main reason for the study, so the results cannot be verified.

Future studies should try to include teenagers from different regions of Spain in the sample, going beyond the current regional setting. This would improve the findings’ generalizability and provide a more thorough comprehension of life happiness in many sociocultural contexts. Furthermore, adding further waves of data collection would make it easier to create longitudinal research on adolescent well-being and enable the assessment of long-term temporal stability. In addition to expanding the geographic and temporal scope, it would be very beneficial to incorporate other factors like social media use, academic achievement, and socioeconomic position, all of which have been strongly connected to teenage life satisfaction. More in-depth understanding of certain risk and protective factors in the mental health of children and adolescents may be possible by investigating the ways in which these variables interact with subjective well-being.

Moreover, examining metric invariance across further subgroups—such as educational stage or age—may help tailor well-being assessments to different developmental periods, increasing the scale’s diagnostic precision. In the long term, validating brief and stable tools like the Satisfaction with Life Scale (SWLS) may support the creation of screening protocols and personalized interventions in educational and community settings. These insights could inform the design of targeted mental health policies and programs that foster comprehensive development and emotional resilience in adolescents, particularly in underrepresented or vulnerable populations.

In practice, schools and educational authorities might use short, validated instruments like the SWLS to conduct periodic LS evaluations in order to identify children who may be at risk of poor well-being and send them to school professionals or mental health specialists. Additionally, preparing educators to analyze and use these findings may facilitate the incorporation of emotional health into lesson planning, particularly in high-stress settings [79,80]. Using brief, dependable measures would enable cost-effective large-scale monitoring in underserved populations or remote schools where access to psychological help is limited.

Moreover, by integrating LS metrics into youth development initiatives, public health authorities might direct funding toward areas or populations with consistently low satisfaction ratings. Using information from LS evaluations to design focused workshops on social belonging, stress management, and emotional regulation would allow for evidence-based interventions centered on the real needs of teenagers.

## 5. Conclusions

A tool widely validated in other contexts that allows the analysis of LS levels in adolescents was assessed in a sample of students from the Autonomous Community of Extremadura (Spain). The results suggested a monofactorial structure composed of five items, without the need for error correlation and reporting acceptable goodness-of-fit indices. On the other hand, the instrument showed total invariance for gender and configural invariance for the students’ place of residence. Likewise, reliability and temporal stability indicators were also satisfactory within the specific sample. Nevertheless, these findings should be interpreted with caution due to the regional nature of the sample, which may limit the generalizability of the results.

## Figures and Tables

**Table 1:** Sample characteristics.

Variable	Categories	N	%
Sex	Boy	200	50
	Girl	200	50
Demographic location	Rural	180	45
	Urban	220	55
**Variable**		**M**	**SD**
Age		13.12	1.94

N: number; %: percentage; SD: standard deviation; M: mean.

**Table 2:** Descriptive statistics and differences on SWLS scale.

Item	Total		Men		Women				Rural		Urban			
	Me	IQR	Me	IQR	Me	IQR	*p*	g	Me	IQR	Me	IQR	*p*	g
1	4.00	2.0	4.00	2.0	4.00	1.0	0.01	0.18	4.00	2.0	4.00	2.0	0.93	0.04
2	5.00	1.0	5.00	1.0	4.00	1.0	0.03	0.22	4.00	1.0	5.00	1.0	0.46	0.05
3	5.00	1.0	5.00	1.0	5.00	1.0	<0.01	0.22	5.00	1.0	5.00	1.0	0.53	0.01
4	4.00	1.0	5.00	1.0	4.00	1.0	0.05	0.13	5.00	1.0	4.00	1.0	<0.01	0.23
5	4.00	2.0	4.00	2.0	4.00	2.0	0.01	0.20	4.00	2.0	4.00	2.0	0.23	0.13
SWLS	4.20	1.0	4.40	1.0	4.00	1.0	<0.01	0.26	4.20	1.0	4.20	1.0	0.84	0.01

Note: Me = median value; IQR = interquartile range; g = Hedges’ g; SWLS: Satisfaction with Life Scale.

**Table 3:** Eigenvalues and variance proportion for the items of the scale.

Items	Eigenvalues	Proportion of Variance
In most ways, my life is close to my ideal.	3.37	0.67
2. The conditions of my life are excellent.	0.52	0.10
3. I am satisfied with my life.	0.48	0.09
4. So far, I have gotten the important things I want in life.	0.45	0.09
5. If I could live my life over, I would change almost nothing.	0.18	0.04

**Table 4:** Unrotated loading matrix.

Items	Load	Communality
In most ways, my life is close to my ideal.	0.73	0.53
2. The conditions of my life are excellent.	0.79	0.62
3. I am satisfied with my life.	0.92	0.84
4. So far, I have gotten the important things I want in life.	0.70	0.49
5. If I could live my life over, I would change almost nothing.	0.72	0.52

**Table 5:** Factor loadings of different subgroups.

Item	Men	Women	Rural	Urban	Total
	Loadings (*R*^2^)	Loadings (*R*^2^)	Loadings (*R*^2^)	Loadings (*R*^2^)	Loadings (*R*^2^)
1	0.58 (0.34)	0.59 (0.35)	0.60 (0.36)	0.59 (0.35)	0.58 (0.34)
2	0.74 (0.55)	0.81 (0.65)	0.73 (0.54)	0.81 (0.65)	0.77 (0.60)
3	0.84 (0.70)	0.88 (0.78)	0.81 (0.65)	0.90 (0.81)	0.76 (0.75)
4	0.61 (0.37)	0.59 (0.34)	0.61 (0.38)	0.60 (0.36)	0.59 (0.35)
5	0.58 (0.34)	0.65 (0.43)	0.73 (0.53)	0.56 (0.31)	0.63 (0.40)

Note: All standardized factor loadings estimated were statistically significant (*p* < 0.01).

**Table 6:** Metric invariance for gender and demographic location.

Gender								
Model	χ^2^	df	CMIN/DF	CFI	NFI	RMSEA	RMSR	ΔCFI
Unconstrained	19.35	8	2.42	0.984	0.97	0.06	0.03	
Configural Invariance	23.12	12	1.93	0.984	0.97	0.05	0.04	<0.01
Metric Invariance	30.27	17	1.78	0.982	0.96	0.04	0.04	<0.01
Scalar Invariance	31.51	18	1.75	0.981	0.96	0.04	0.04	<0.01
**Demographic Location**								
**Model**	**χ^2^**	**df**	**CMIN/DF**	**CFI**	**NFI**	**RMSEA**	**RMSR**	**ΔCFI**
Unconstrained	19.93	8	2.49	0.984	0.97	0.06	0.03	
Configural Invariance	27.79	12	2.32	0.979	0.96	0.06	0.05	<0.01
Metric Invariance	39.91	17	2.35	0.969	0.95	0.06	0.05	>0.01
Scalar Invariance	39.91	18	2.22	0.967	0.95	0.05	0.05	<0.01

**Table 7:** Temporal stability.

SWLS	M (SD)	ICC (95% CI)	SEM	MDC (%)	MDC90 (%)	MDC95 (%)
Time 1	4.06 (0.77)	0.70 (0.62, 0.76)	0.39	0.88 (13.32)	0.91 (21.92)	1.08 (26.12)
Time 2	4.24 (0.65)					

Note: M = mean value; SD = standard deviation; ICC = intraclass correlation coefficient; SEM = standard error of measurement; MDC = minimal detectable change.

## Data Availability

The datasets are available through the corresponding author upon reasonable request. The data are not publicly available due to privacy.

## Appendix A

**Table A1:** Escala de Satisfacción con la Vida (SWLS).

En la mayoría de los aspectos mi vida es como yo quiero que sea
2. Las circunstancias de mi vida son muy buenas
3. Estoy satisfecho con mi vida
4. Hasta ahora he conseguido de la vida las cosas que considero importantes
5. Si pudiera vivir mi vida otra vez no cambiaría casi nada

## Appendix B

**Table A2:** Escala de Satisfacción con la Vida para Estudiantes (SLSS).

Mi vida va bien
2. Mi vida es perfecta
3. Me gustaría cambiar muchas cosas de mi vida
4. Te gustaría tener otro tipo de vida
5. Tengo una buena vida
6. Tengo lo que quiero en la vida
7. Mi vida es mejor que la mayoría de los chichos/as de mi edad
